# Supplementary material for: Frailty index as a predictor of all-cause and cause-specific mortality in a Swedish population-based cohort
Source: Aging (Albany NY). 2017 Dec 19;9(12):2629–46. doi: 10.18632/aging.101352 (PMC5764396; doi:10.18632/aging.101352)
Supplement: Supplementary file 1 [file aging-09-2629-s001.pdf]

SUPPLEMENTARY MATERIAL

SUPPLEMENTARY METHODS

Multiple imputation

After selection of the 42 items (all with <10% missing data points) that were included in the frailty index (FI) (Supplementary Table 1), we followed the practices previously used by the Rockwood group [1, 2] in excluding all individuals with ≥20% missing data (n=160, 9.8%) across the FI items and then performed multiple imputation (MI) to replace missing data for 400 individuals. Inspection of the missingness pattern graphics revealed that the values were missing at random i.e., no monotonicity was observed. Constraints (min, max, increment/rounding) for the imputed values were set to match the scoring of the given FI item (Supplementary Table 1). Five rounds of imputations were performed and the pooled mean from the simulations was used as the final value for each missing data point. After that all the item scores were summed and the FI was calculated by dividing the sum by 42. As a sensitivity analysis, we performed a Cox regression analysis for all-cause mortality using age, smoking status and FI as covariates and stratifying by sex first for those individuals with no missing data in the FI items (n=1077) and then using imputed data set (n=1477). Almost identical estimates were obtained using these data sets (data not shown). Hence, the dataset with the imputed data was used in this study.

Cause-specific mortality analyses

Two approaches were taken to analyze the relationship between the FI and cause-specific mortality: a cause-specific hazards model (CHR) based on the "standard" Cox regression and a subdistribution hazards model. The latter is also a Cox model but instead of the hazard ratio (HR) it utilizes a subdistribution hazard ratio (SHR) derived from for the cumulative incidence function (CIF) by Fine and Grey [3]. Heuristically, the SHR model CIF for the kth cause of death can be defined as: $CIF_k(t) = \Pr(T \leq t, D = k)$, where D denotes the cause of death of interest [4]. The occurrence of D precludes the subsequent occurrence of deaths due to the other causes and the $CIF_k(t)$ denotes the probability of experiencing the kth event before time t and before the occurrence of death due to the other causes. The SHR thus represents a ratio in a "non-existing" population including also those who experienced death due to the other causes. This approach is more suited for clinical risk predictions where estimating the absolute risk is of more interest than in settings addressing etiological questions and the instantaneous risks [4, 5]. On the other hand, unlike the CHR model, the SHR model has the advantage that it does not assume independent and noninformative censoring. That is, information about a subject's risk of experiencing one type of event should provide no information about the subject's risk of experiencing the other type of event. However, human biology often suggests at least some level of dependence between competing risks, yet there is no explicit way to test this assumption in a given data set [4].

Analogously to the all-cause mortality analysis, the cause-specific hazards and subdistribution hazards were modeled separately for men and women, considering deaths due to cancer, CVD, dementia and other causes as the competing risks. If an association between the FI and a risk of interest was observed in the CHR model, a sensitivity analysis for the consensus classification (Supplementary Table 3) was performed by excluding or including individuals with multiple causes of death. That is, for cancer mortality we excluded individuals who also had CVD as a cause of death as cancer overrode CVD in the consensus classification, whereas for CVD mortality we included those who had also cancer as a cause of death. In the case of CVD and cancer mortality, we wished to examine if the association was independent of presence of these diseases and thus performed an additional adjustment for the CVD and cancer status in the CHR models, respectively. Of the 187 women who died of CVD, 118 had CVD at the study baseline whereas of those 89 women who died of cancer 8 had cancer at the study baseline. Having any of the following disorders were considered as CVD: angina pectoris, myocardial infarction, stroke, high blood pressure, claudication, phlebitis, circulation problems in limbs and thrombosis.

SUPPLEMENTARY REFERENCES

1. Rockwood K, Song X, Mitnitski A. Changes in relative fitness and frailty across the adult lifespan: evidence from the Canadian National Population Health Survey. CMAJ. 2011; 183:E487-94.

2. Theou O, Brothers TD, Mitnitski A, Rockwood K. Operationalization of frailty using eight commonly used scales and comparison of their ability to predict all-cause mortality. J Am Geriatr Soc. 2013; 61:1537-51.

3. Fine JP,Gray RJ. A Proportional Hazards Model for the Subdistribution of a Competing Risk. Journal of the American Statistical Association. 1999; 94:496-509.

4. Austin PC, Lee DS, Fine JP. Introduction to the Analysis of Survival Data in the Presence of Competing Risks. Circulation. 2016; 133:601-609.

5. Noordzij M, Leffondré K, van Stralen KJ, Zoccali C, Dekker FW, Jager KJ. When do we need competing risks methods for survival analysis in nephrology? Nephrology Dialysis Transplantation. 2013; 28:2670-2677.

SUPPLEMENTARY TABLES

Supplementary Table 1. List of the 42 items included in the frailty index and their scoring.

Item	Scoring
Hearing status	Perfect=0, Good=0.25, Pretty Good=0.5, Bad=0.75, Deaf or almost deaf=1
Vision status	Perfect=0, Good=0.25, Pretty Good=0.5<br>Bad=0.75, Blind or almost blind=1
Health prevents from doing things normally would like to do	No=0, Somewhat=0.5, Yes=1
Self-reported general health	Good=0, Mediocre=0.5, Bad=1
Cancer or leukemia	No=0, Yes=1
Rheumatoid arthritis	No=0, Yes=1
Arthritis	No=0, Yes=1
Chronic bronchitis or emphysema	No=0, Yes=1
Cataracts	No=0, Yes=1
Chest pain	No=0, Yes=1
Circulation problems in arms or legs	No=0, Yes=1
Persistent cough	No=0, Yes=1
Diabetes	No=0, Yes=1
Goiter or other gland problems	No=0, Yes=1
Heart failure	No=0, Yes=1
Hypertension	No=0, Yes=1
Kidney disease	No=0, Yes=1
Brittle bones	No=0, Yes=1
Sciatica	No=0, Yes=1
Anemia	No=0, Yes=1
Cerebral hemorrhage or blood clot in brain	No=0, Yes=1
Dizziness	No=0, Yes=1
Gastric ulcer	No=0, Yes=1
Allergies/allergic manifestations	No=0, Yes=1
Asthma	No=0, Yes=1
Shower and bathe[1]	No problem=0, Needs help=0.5, Cannot=1
Get in and out of bed[1]	No problem=0, Needs help=0.5, Cannot=1
Dress and undress[1]	No problem=0, Needs help=0.5, Cannot=1
Self-grooming[1]	No problem=0, Needs help=0.5, Cannot=1
Walking[1]	No problem=0, Needs help=0.5, Cannot=1
Trouble getting to toilet in time[1]	No=0, Yes=1
Travel further distances[2]	Can travel alone=0, Can go by taxi=0.5,<br>Needs helper, special assistance or doesn't travel=1
Housework[2]	No problems=0, Needs help=0.5, Doesn't do=1
Prepare meals[2]	Can plan/prepare=0, Can heat up=0.5,<br>Doesn't cook=1
Manage medications[2]	No problems=0, Needs help=0.5, Doesn't do=1
Manage money[2]	No problems=0, Needs help=0.5, Doesn't do=1
Use telephone[2]	Can look up numbers and dial=0<br>Needs help or doesn't use phone=1

Grocery shopping[2]	Can shop=0, Needs help=0.5, Doesn't shop=1
Feeling lonely[3]	Never, almost never, rather seldom=0<br>Quite often, always, almost always=1
Feeling depressed[3]	Never, almost never or rather seldom=0<br>Quite often, always, almost always=1
Consider oneself happy and carefree	No=1, Yes=0
Usually feels tired	No=0, Yes=1

Note: [1]from the instrument of Basic Activities of Daily Living, [2]from the instrument of Instrumental Activities of Daily Living, [3] from the Center for Epidemiologic Studies Depression Scale

Supplementary Table 2. ICD codes used to classify the competing risks.

	ICD-7	ICD-8	ICD-9	ICD-10	Surgical code
**Dementia**	304-306	290	290	F00-F03	
		293.0-293.1	294B	G30	
			331A-331C	G311	
			331X	G318A	
				F051	
**Non-stroke CVD**	420	410-414	410-414	I20-I25	984
	450	440	440	I70	3068
	453.33	443.90	443X	I73.9	3080
					3127
					3141
					3158
					FNC
					FND
					FNE
					FNG00
					FNG02
					FNG05
**Stroke**	330	430-431	430-431	I60-I61	
	331.00-331.01	433-434	434	I63-I64	
	331.09	436	436		
	331.99				
	332.00-19				
	332.29				
	334.00-98				
**Cancer**	140-205	140-209	140-208	C00-C97	
				B21	

Note: Non-stoke CVD and Stroke were considered as CVD-mortality
Abbreviations: ICD, International Classification of Diseases; CVD, cardiovascular disease

Supplementary Table 3. Consensus classification for the competing risks when more than one cause of death was recorded.

Cancer	CVD	Dementia	Consensus	Count in men	Count in women
-	-	+	Dementia	20	58
-	+	-	CVD	149	159
+	-	-	Cancer	124	73
-	+	+	CVD	11	28
+	+	-	Cancer	15	12
+	-	+	Cancer	3	3
+	+	+	Cancer	1	1

Abbreviations: CVD, cardiovascular disease

Supplementary Table 4. Causes of deaths included classified as other-cause mortality.

	Men	Women	All
Blood	0	1	1
Circulatory	23	73	96
Congenital	0	1	1
Digestive	8	15	23
Endocrine/metabolic	5	6	11
Genitourinary	2	4	6
Infections	4	6	10
Injuries	7	12	19
Musculoskeletal	2	2	4
Neurological	1	6	7
Psychiatric	3	1	4
Respiratory	26	25	51
Skin	0	2	2
Tumour (non-cancer)	1	2	3
Other	7	13	20
Total	89	169	258

SUPPLEMENTARY FIGURES

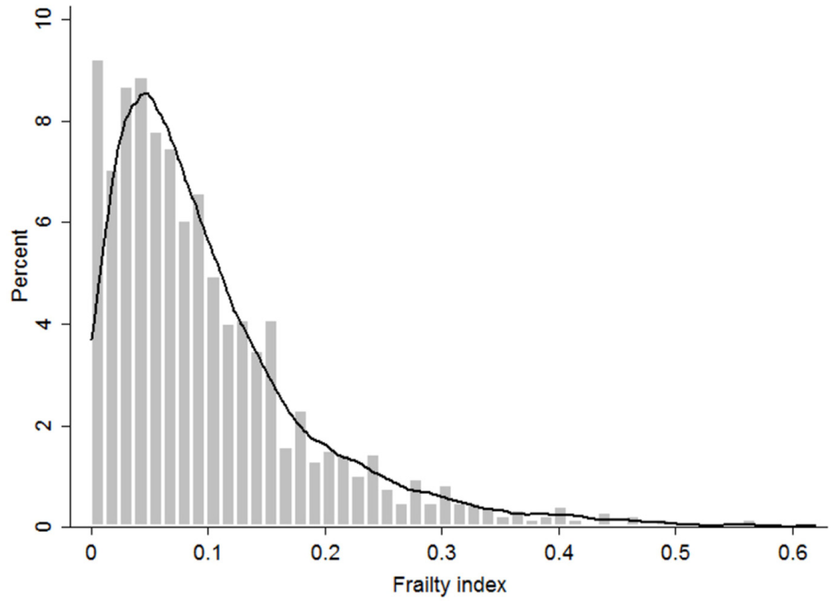

Supplementary Figure 1. Distribution of the FI in our sample. The line represents a kernel density plot.

A

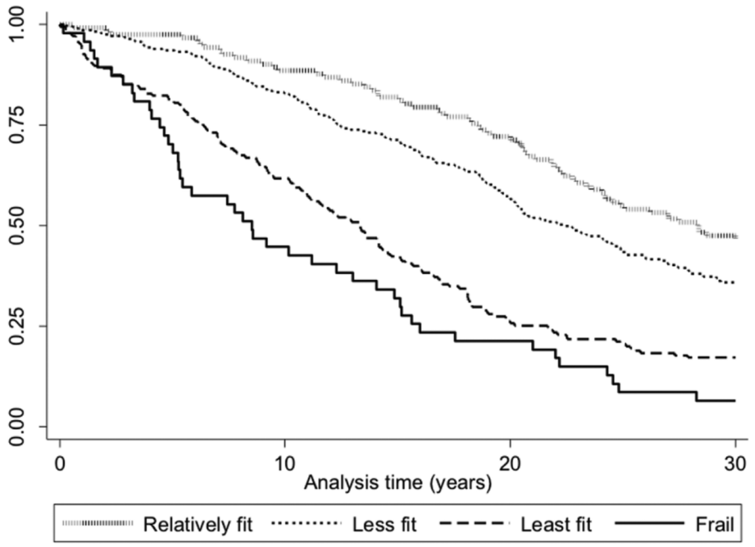

B

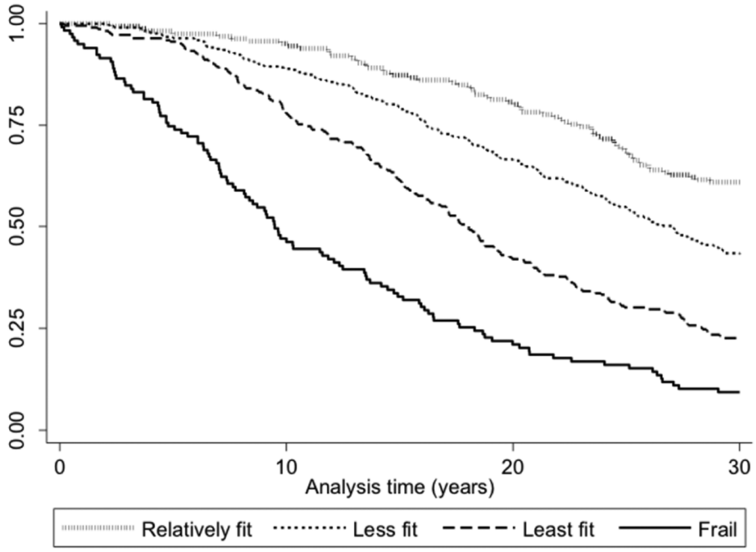

Supplementary Figure 2. Kaplan-Meier survival probabilities according to the categorized FI index in men (**a**) and women (**b**).

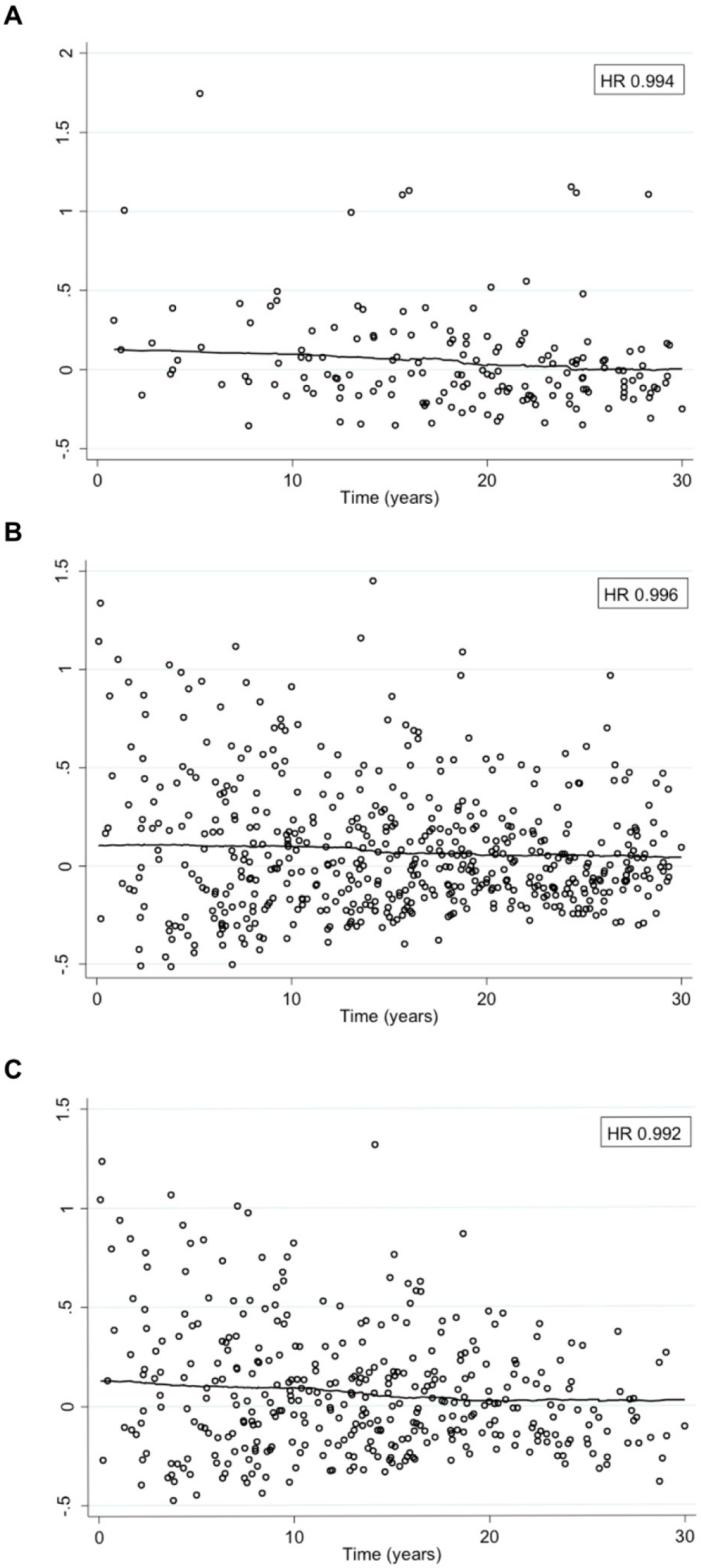

Supplementary Figure 3. The Schoenfeld residual plots for the testing of the proportional hazards assumption in the young men (**a**), all women (**b**) and young women (**c**). Hazard ratios (HRs) for the time-varying coefficient of the frailty index are presented top right.
